# Epstein−Barr virus-encoded EBNA2 alters immune checkpoint PD-L1 expression by downregulating miR-34a in B-cell lymphomas

**DOI:** 10.1038/s41375-018-0178-x

**Published:** 2018-06-26

**Authors:** Eleni Anastasiadou, Dina Stroopinsky, Stella Alimperti, Alan L Jiao, Athalia R Pyzer, Claudia Cippitelli, Giuseppina Pepe, Martina Severa, Jacalyn Rosenblatt, Marilena P Etna, Simone Rieger, Bettina Kempkes, Eliana M Coccia, Shannan J Ho Sui, Christopher S Chen, Stefania Uccini, David Avigan, Alberto Faggioni, Pankaj Trivedi, Frank J Slack

**Affiliations:** 1 Harvard Medical School Initiative for RNA Medicine, Department of Pathology, Beth Israel Deaconess Medical Center, Harvard Medical School, Boston, MA USA; 2 Department of Hematology, Beth Israel Deaconess Medical Center, Harvard Medical School, Boston, MA USA; 3 The Wyss Institute for Biological Inspired Engineering at Harvard, Harvard University, Boston, MA USA; 4 Department of Clinical and Molecular Medicine, Sant’Andrea Hospital, Sapienza University, Rome, Italy; 5 Department of Infectious Diseases, Istituto Superiore di Sanità, Rome, Italy; 6 Helmholtz Zentrum München, Deutsches Forschungszentrum für Gesundheit und Umwelt (GmbH), Marchioninistraße 25, 81377 Munich, Germany; 7 Bioinformatics Core, Harvard T. H. Chan School of Public Health, Boston, MA 02115 USA; 8 Department of Experimental Medicine, Sapienza University, Viale Regina Elena 324, 0161 Rome, Italy

**Keywords:** Cancer, Tumour virus infections

## Abstract

Cancer cells subvert host immune surveillance by altering immune checkpoint (IC) proteins. Some Epstein−Barr virus (EBV)-associated tumors have higher Programmed Cell Death Ligand, PD-L1 expression. However, it is not known how EBV alters ICs in the context of its preferred host, the B lymphocyte and in derived lymphomas. Here, we found that latency III-expressing Burkitt lymphoma (BL), diffuse large B-cell lymphomas (DLBCL) or their EBNA2-transfected derivatives express high PD-L1. In a DLBCL model, EBNA2 but not LMP1 is sufficient to induce PD-L1. Latency III-expressing DLBCL biopsies showed high levels of PD-L1. The PD-L1 targeting oncosuppressor microRNA miR-34a was downregulated in EBNA2-transfected lymphoma cells. We identified early B-cell factor 1 (EBF1) as a repressor of miR-34a transcription. Short hairpin RNA (shRNA)-mediated knockdown of EBF1 was sufficient to induce miR-34a transcription, which in turn reduced PD-L1. MiR-34a reconstitution in EBNA2-transfected DLBCL reduced PD-L1 expression and increased its immunogenicity in mixed lymphocyte reactions (MLR) and in three-dimensional biomimetic microfluidic chips. Given the importance of PD-L1 inhibition in immunotherapy and miR-34a dysregulation in cancers, our findings may have important implications for combinatorial immunotherapy, which include IC inhibiting antibodies and miR-34a, for EBV-associated cancers.

## Introduction

Among non-Hodgkin lymphoma (NHL), more than 95% of endemic BLs are associated with Epstein−Barr virus (EBV). Diffuse large B-cell lymphomas (DLBCLs) constitute about 30% of all NHLs, of which about 10% are EBV associated in immunocompetent patients [1]. Its high frequency makes DLBCL one of the most common cancers in adults [2]. It is noteworthy that the annual global number of cases of EBV-positive DLBCLs supersede the total number of BLs. Additionally, EBV is the cause of lymphomas arising in immunocompromised individuals such as AIDS and transplant patients [3]. This clearly suggests that EBV’s ability to cause cancer lies in its capacity to evade host immune surveillance.

EBV generally establishes one of the following four forms of latency, depending upon the phenotype and the transcription factor repertoire of the infected cells [4]. A complete lack of any virally encoded latent gene expression program as that seen in the resting memory B cell is called latency 0. The expression of the virally encoded EBNA1 and EBERs represents type I latency. EBV-infected normal B lymphocytes express type I latency in vivo [5]. Under pathological conditions, the viral latent-gene expression varies in different tumors. The phenotypically representative BL and corresponding cell lines express EBNA1 and LMP2A. When these lines drift towards an immunoblastic phenotype, the viral gene expression is expanded to all growth transformation proteins, EBNA1 to -6 and LMP1, -2A, and -2B. Collectively, this is known as the type III program. The viral latent-gene expression observed in NPC and Hodgkin lymphoma is of intermediate type II latency (LMP1+EBNA2−) [6].

The ability of EBV to transform normal B lymphocytes into permanently growing lymphoblastoid cell lines (LCLs) is attributed to its latent proteins. Among these, LMP1 and EBNA2 have been extensively studied [7, 8]. In particular, it is known that EBNA2 is sine qua non for the virus to transform B cells [9]. Indeed, in keeping with its importance in transformation, EBNA2 expression ensues early after EBV infects naive B cells [10]. This viral protein is also a potent activator of transcription such as CD23 and C-myc [11, 12] but can also negatively regulate genes like *BCL6* and *Ig* [13, 14]. It is a functional homolog of intracellular (Ic) Notch, although they are not interchangeable [15, 16]. It does not bind directly to DNA but activates transcription of many target genes by binding to the transcription factor, RBP-Jk [17]. EBNA2 colocalizes with another B-cell-specific DNA binding transcription factor, EBF1 [16], which is essential for the commitment and maintenance of B-cell transcription program [18, 19].

Immune checkpoints (IC) regulate T-cell responses to maintain self-tolerance. They deliver costimulatory and coinhibitory signals to T cells [20]. PD-L1, mainly expressed by antigen-presenting cells engages its receptor PD-1 on T cells, to provide a growth inhibitory signal. Different tumors express high PD-L1 to evade immune recognition and consistently, inhibition of PD-1/PD-L1 and other IC molecules have become important targets of cancer immunotherapy [21].

MicroRNAs (miRNAs) are small noncoding RNAs that post-transcriptionally regulate gene expression [22, 23]. The miR-34 family members are transcriptionally induced by p53 [24]. They suppress transcription of genes important in cell cycle progression, antiapoptotic functions, and regulation of cell growth. Expression of miRNAs is altered in a broad range of cancers, with frequent downregulation of both p53 and miR-34 [25, 26]. The latter is downregulated in chronic lymphocytic leukemia and acute myeloid leukemia (AML) [27, 28]. Interestingly, the IC protein, PD-L1, has been shown to be a validated target of miR-34a [29].

Based on gene expression, DLBCLs are divided into two broad categories, the germinal center (GC) type and the activated B-cell type (ABC) or the non-GC type [30]. The overall survival rates in the non-GC (ABC) DLBCL patients are poor [31–34]. EBV is associated more frequently with the non-GC DLBCLs [2], which generally express high levels of PD-L1 [31]. Both EBV associated and high PD-L1 expressing non-GC DLBCLs have a very poor prognosis [31, 35]. In other hematological malignancies, like Hodgkin Lymphoma (HL), high PD-L1 expression has been reported due to either selective amplification of the PD-L1 locus on chromosome 9p24.1 or EBV infection [36]. These two modes of PD-L1 upregulation are mutually exclusive [37]. It was also shown that LMP1 expression induced PD-L1 promoter activity in B cells [37]. In addition, more than 70% of post-transplant lymphoproliferative disorders, of which EBV is the cause, express PD-L1 [37]. In DLBCL, Kwon et al. [32] observed that PD-L1 expression was positively correlated with EBV’s presence in ABC type DLBCL.

Although the presence of EBV is correlated with higher expression of PD-L1 both in HL and DLBCLs, it is not clear if and how the virus is responsible for an increased PD-L1 expression and if this applies to other lymphomas like BLs, as well. While LMP1 has been implicated in induction of PD-L1 in HEK293 cells [37] or in epithelial cells [38], it is not known if other EBV encoded genes like EBNA2 can regulate PD-L1 in a more frequent cellular setting and natural reservoir for EBV, such as B cells. In this study, we set out to investigate if EBNA2, which is indispensable for EBV’s ability to transform B cells, has any effect on PD-L1 and if this involves regulation of cellular miRNAs.

## Methods

### Cells

Mutu I and Mutu III, Daudi, Jijoye are EBV-positive BLs. LCL is an EBV-positive cell line. OMA4 [39], DG75, and BL41 are EBV-negative BLs. U2932, SUDHL5 are EBV-negative GC-type DLBCLs. ER/EB 2.5 is an estradiol-inducible EBNA2 carrying cell line [40]. The details of the cell lines infected with recombinant EBV [14, 41–43] and EBNA2/ LMP1 transfectants [14, 44] have been described previously.

### Infection with a recombinant EBV strain

The recombinant strain of Akata EBV [45] was a kind gift from Prof. Kenzo Takada (Hokkaido University, Sapporo, Japan). The induction of lytic replication, virus production by engaging IgG with corresponding antibodies and infection procedure has been described in detail by us previously [14, 41, 42]. The supernatant containing recombinant EBV was used to infect EBV-negative U2932, SUDHL5, OMA4, and DG75 cells.

### EBNA2 and LMP1 transfection and selection

An EBNA2 expression vector J144-C1, the expression vector for LMP1 J132-G5 and the corresponding vector control pSV-MPA GPT (a kind gift from Prof. Lars Rymo, Gothenburg University, Sweden) were individually transfected into U2932 DLBCL cells by electroporation. The transfection and selection details have been described by us previously [14, 46]. BL41K3 cells transfected with estrogen-inducible EBNA2 were treated with 1 µM estradiol to induce EBNA2 expression [44].

### Immunoblotting

EBNA2 and LMP1 expression was verified by monoclonal antibodies PE2 (Kindly provided by Dr. Martin Rowe, Birmingham University Medical School) [47] and S12 monoclonal antibodies (a kind gift from the late Dr. David Thorley-Lawson, Tufts University, Boston, USA), respectively. β-actin antibodies were purchased from Sigma. PD-L1 (E1L3N, cat# 13684) and p21 (#2947) and BCL2 (#15071) were purchased from Cell Signaling. Further details of the method are in supplementary information.

### Quantitative RT-PCR (qRT-PCR)

Total RNA from cell lines was isolated using Direct-zol RNA MiniPrep Plus kit (Zymo Research) according to the vending company’s instructions. The integrity of RNA was routinely checked using 1% agarose gel and RNA quantification was estimated with a DS-11 spectrophotometer (DeNovix) [48]. The cDNA synthesis for mature miR-34a was performed according to the manufacturer’s instructions (miScript II RT Kit, Qiagen). For verification of pre-miR-34a expression, reverse transcription qPCR was performed. Further details of the method can be found as supplementary information.

### Knockdown of miR-34a and miR-34 mimic transfection

The U2932 vector control or EBNA2-expressing clones were transfected with 50 nM of miR-34a inhibitors (mirVana), mimic miR-34a oligonucleotides or mimic controls, purchased from Ambion. The compounds were delivered into the cells with DharmaFect Duo transfection reagent (GE Dharmacon). After 48 h, the cells were harvested for total RNA and protein extraction.

### miR-34a promoter and biosensor luciferase reporters

To investigate the effect of EBNA2 on miR-34a promoter, 50,000 EBNA2-expressing U2932 and BL41 cells were seeded in triplicates in a 96-well plate. The cells were co-transfected with 1 µg/µl of pRL-TK luciferase control reporter (Promega) and 0.5 µg/µl miR-34aP luciferase reporter, which carries the wild-type miR-34a promoter (Addgene plasmid # 50827). After 48 h, the cells were harvested and lysed in 80 µl passive lysis buffer (Promega). Firefly and Renilla luciferase activity was detected by GloMax explorer luminometer (Promega). To verify miR-34a mimic transfection efficiency in U2932 and EBNA2 clone 1, 50 nM miR-34a mimics were co-transfected with 20 ng of miR-34a or miR-34a mismatch biosensors. Both biosensors were generated by cloning the reverse miR-34a or miR-34a mismatched complementary sequence cloned downstream of the Renilla promoter in the psiCheck-2 dual luciferase reporter plasmids (Promega), as previously described [49, 50]. After 48 h post-transfection, the cells were lysed in 80 µl passive lysis buffer (Promega). Firefly and Renilla luciferase activity was detected by GloMax explorer luminometer (Promega).

### PD-L1 luciferase reporters and activity

PD-L1 3′UTR Luciferase reporter construct was made as follows. The full-length PD-L1 3′UTR (2674 bp) (ref|NM_014143.3| Homo sapiens CD274 molecule (CD274), transcript variant 1, mRNA) was PCR amplified from human genomic DNA (Thermo Fisher #4312660), in three separate fragments. Fragment 1 was generated with primers F: GAGACGTAATCCAGCATTGG and R1: CTGAGGTCTGCTATTTACTGG; Fragment 2 was generated with primers F1: CCAGTAAATAGCAGACCTCAG and R2: GACTAGATTGACTCAGTGCAC; Fragment 3 was generated with primers F2: GTGCACTGAGTCAATCTAGTC and R: TAACTTTCTCCACTGGGATG. The three fragments were connected by overlap PCR, with forward primer: actcgagGAGACGTAATCCAGCATTGG (containing a *Xho*I site, underlined) and reverse primer: agcggccgcTAACTTTCTCCACTGGGATG (containing a *Not*I site, underlined). The full-length PD-L1 3′UTR was cloned into the Psicheck2 vector between the *Xho*I and *Not*I sites downstream of Renilla luciferase, and fully verified by sequencing. Further details are enclosed in the supplementary information.

### Site-directed mutagenesis of PD-L1 3′UTR

Point mutations were introduced into the miR-34a seed sequence of 3′UTR of PD-L1 cloned in Psicheck-2 vector according to the QuikChange site-directed mutagenesis kit (Agilent Technologies). The mutagenic primers containing the desired mutation in the miR-34a seed sequence of the 3′UTR of PD-L1 were: Forward primer: 5′-GAAGCAACTGCTACGAACGTTCATTCATATG-3′ and the reverse primer: 5′-CATATGAATGAACGTTCGTAGCAGTTGCTTC-3′. The miR-34a seed sequence in the wild type 3′-UTR of PD-L1 is in bold letters: 5′**-**GAAGCAACTGCTAC**TGCCT**TTCATTCATATG-3′. **TGCCT** was mutated to **GAACG**. The mutated seed sequence was verified by sequencing.

### EBF1 knockdown

Knockdown of EBF1 was obtained by transduction of U2932 and its EBNA2 expressors with pLK0.1 lentiviral vectors, which carry shEBF1 and the corresponding control shRNA (TRC Human EBF1 shRNA, Clone ID: TRCN0000013831 and Plko.1-emptyT control TRCN0000208001, Open Biosystems, Dharmacon). Cells were transduced as described below and were selected with 1.5 µg/ml puromycin for 10 days and used for further experiments.

### Lentivirus transduction

The cell lines U2932 MPA vector and U2932 EBNA2 were transduced with pLL3.7_hsa-miR-34a (Addgene plasmid # 25791) and pLL3.7 control vector (Addgene plasmid # 11795). For the production of lentiviruses, viable and confluent HEK293T cells were transfected (Fugene6, Promega) with the transfection mixture composed of 10 µg of pLL3.7_hsa-miR-34a or pLL3.7 vector control along with 5 µg pMD2.G envelope plasmid (Addgene plasmid # 12259) and 5 µg psPAX2 packaging plasmid (Addgene plasmid #12260). The method is further detailed as supplementary information.

### Standard mixed lymphocyte reaction (MLR)

PBMCs were isolated from the blood of healthy donors using Ficoll-Paque separation media (GE Healthcare) and were seeded in 24-well non-tissue culture-treated plates (Falcon, Fisher, Pittsburgh, PA, USA), previously coated with anti-CD3 (clone-UCHT1; Pharmingen, San Diego, CA, USA) and anti-CD28 (clone-CD28.2; Pharmingen, San Diego, CA, USA) at the concentration of 1 μg/ml in phosphate-buffered saline (PBS) at 0.4 ml/well overnight at 4 °C.

The day after, the plates were washed in 1× PBS and PBMCs were added to the CD3/CD28-coated wells at a density of 1×10^6^ cells/well and cultured for 72 h, in order to activate the CD4 and CD8 cell population.

One day before seeding the stimulators, 1×10^5^ U2932 MPA vector and U2932 EBNA2 cl-1 were transiently transfected with 50 nM mimic negative control and mimic miR-34a (Ambion) and subsequently irradiated with a sub-lethal dose of 5 Gy for 2 min. The cells were placed in coculture with 1×10^6^ PBMCs. At 72 h post-transfection and after 48 h coculture, all samples were treated for 5 h with GolgiStop™ (BD Biosciences) to block cytokine accumulation in the Golgi complex, for the detection of IFN-γ-producing cells, by flow cytometry. Additional details are in supplementary materials and methods. The entire population of co-cultured cells was stained with FITC mouse anti-human CD8 and Pacific Blue mouse anti-human CD4 (BD Pharmingen) for detection of T cells. The same cells were then permeabilized with cytofix/cytoperm buffer (BD Pharmingen), according to the manufacturer’s instructions. The cells were stained intracellularly with human IFN-γ, R-PE (Invitrogen). A matched isotype control, anti-Human IgG Fc secondary antibody, PE (Invitrogen), was also included in this experiment. Sample acquisition was performed with Gallios Flow Cytometer. The data were analyzed with Kaluza for Gallios Software.

### 3D microfluidic platform for T-cell responses to EBNA2-transfected U2932 DLBCL

The 3D microfluidic chips, polydimethylsiloxane (PDMS, Sylgard 184, Dow-Corning, Midland, Michigan) microfluidic devices were fabricated using soft lithography as described previously [51, 52]. The devices were treated with 0.01% v/v poly-l-lysine and 0.5% v/v gluteraldehyde to promote collagen/fibronectin adhesion. After washing overnight in water, steel acupuncture needles (160 μm diameter, Seirin, Kyoto, Japan) were introduced into the devices and a solution of 2.5 mg/ml type 1 collagen, 1× M199 medium, 1 mM HEPES, 0.1 M NaOH and NaHCO_3_ (0.035% w/v) and 200 ng/ml Fibronectin (Thermo Fisher Scientific, Waltham, MA) was infused into the devices and allowed to polymerize for 40 min at 37 °C. Subsequently, needles were removed to create 160 μm diameter channels within collagen/fibronectin gel and cells were introduced into devices. In coculture experiments, each device was first seeded with 5×10^3^ U2932 EBNA2 cl-1 transduced with the control lentivirus or miR-34a containing lentiviral vector and were incubated for 24 h at 37 °C. Subsequently, 5×10^4^ PBMCs, containing previously activated T cells were added in complete medium (RPM1 1640/10% FBS). The devices were in triplicates and incubated for an additional 48 h before performing immunostaining.

For immunostaining of the cocultures in microfluidic devices, the cells were fixed with 4% PFA for 10 min and washed twice in PBS, permeabilized with 0.1% (v/v) Triton X 100 in PBS for 20 min at room temperature, and treated with a blocking solution (BSA 5% in PBS 0.1% Triton X 100). The devices were incubated with rabbit anti-caspase-3 (Cell Signaling) or mouse anti-CD4 and -CD8 antibodies (1:100 dilution, Biolegend) and kept on a rocking platform O/N at 4 °C. Devices were merged in PBS and left on a rotor O/N, at 4 °C to remove excess antibody. The day after, PBS was removed and Alexa 568-conjugated goat-anti-rabbit IgG (primary abs, caspase-3) and Alexa 647-conjugated goat-anti-mouse IgG (primary abs, CD4 and CD8) secondary antibodies diluted 1:100 in blocking buffer were added per well in each device O/N at 4 °C. Finally, PBS was added in each device and processed to detect caspase-3, CD4 and CD8 staining. The devices were visualized using confocal microscope (LSM 710, Carl Zeiss), and image analysis made by ImageJ by performing a maximum intensity z projection and merging the channels.

### PD-L1 immunohistochemistry and quantitative analysis in biopsies from DLBCL patients

A written informed consent was obtained from all patients involved in the study. The study design was approved by the Institute’s ethics review board. Paraffin sections were immunostained for PDL-1, PD-1, EBNA2, LMP1, MUM-1, CD10, and Bcl6, using an automated immunostainer (DAKO, Glostrup, Denmark). As control for PD-L1 immunostaining, sections from paraffin-embedded human lung carcinoma were used. Further details are in supplementary m&m. For quantitative IHC analysis, the Aperio Imagescope algorithm was used to evaluate both percentage positive cells and intensity of the stained tumor cells in three regions of three clinical samples representing each of the three types of non-GC DLBCL category (EBV neg, EBV+/EBNA− and EBV+/EBNA2+).

## Results

### PD-L1 expression is induced in latency III-expressing BLs, in vitro infected BLs and DLBCLs and EBNA2-transfected cells

**Fig. 1:**
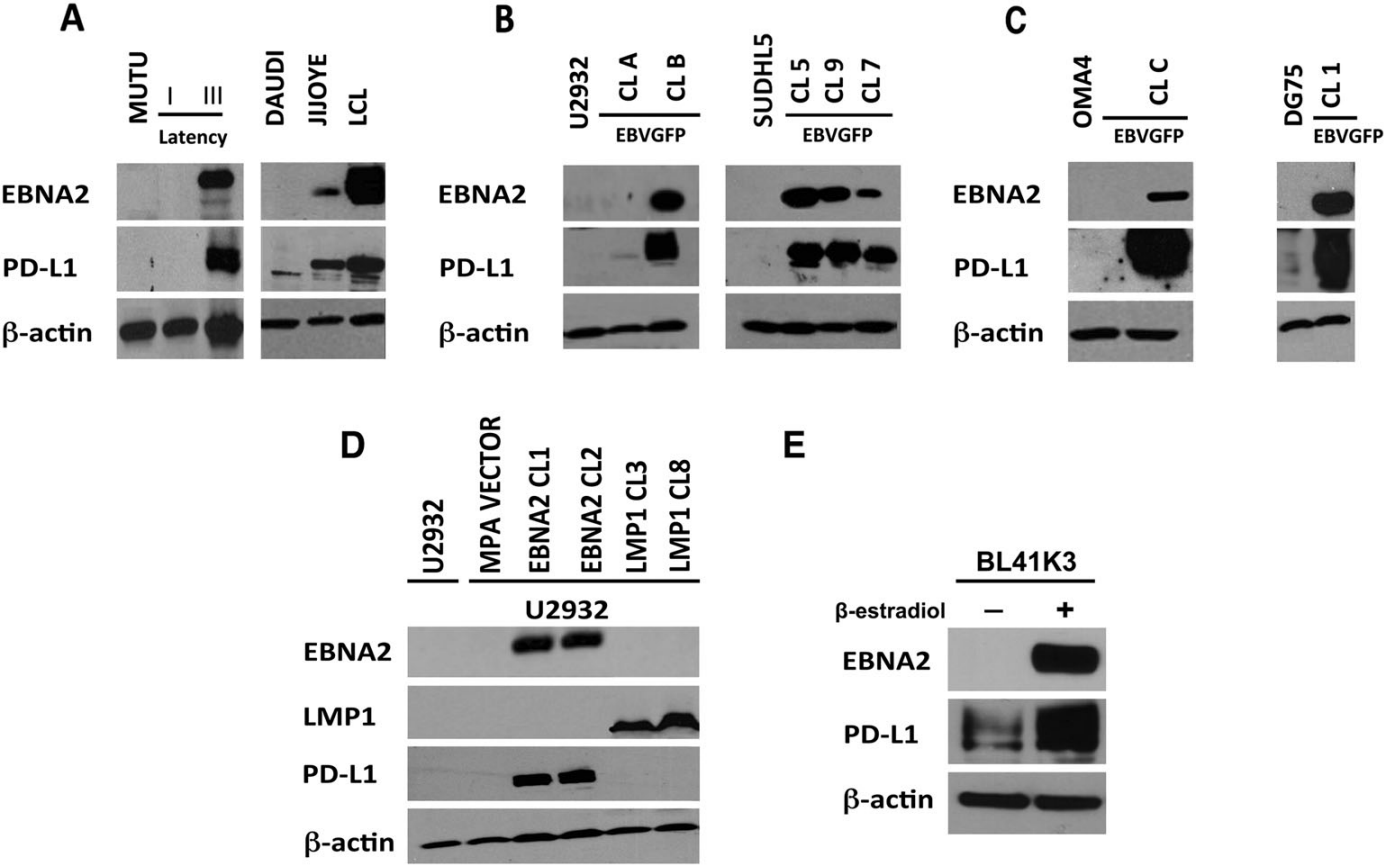
PD-L1 expression in EBV-infected and EBNA2-transfected BL and DLBCL cell lines. **a** EBNA2 and PD-L1 expression in Mutu I (latency I expressor) and its latency III-expressing counterpart. Furthermore, expression of PD-L1 and EBNA2 is shown in two additional BLs with resident viral genomes. Daudi carries an EBNA2-deleted EBV strain and Jijoye is EBNA2-positive BL. **b** Two GC DLBCLs, namely U2932 and SUDHL5, were infected with a recombinant Akata strain of EBV. Total cell lysates were electrophoresed and EBNA2 expression was verified by immunoblotting using PE2 monoclonal antibodies. PD-L1 expression was analyzed using rat monoclonal antibodies. **c** Two BL cell lines, Oma4 and DG75, were infected with the recombinant Akata virus and tested for EBNA2 and PD-L1 expression. β-actin is used as loading control. **d** PD-L1, EBNA2, and LMP1 expression in transfected U2932 DLBCL and **e** BL41 is an EBV-negative BL. The BL41K3 derivatives are estrogen-inducible EBNA2 transfectants. PD-L1, EBNA2, and β-actin expression was analyzed before and after β-estradiol treatment. β-actin is used as loading control

The restricted latency expressor cell line Mutu I [53] did not express PD-L1 while its EBNA2-expressing counterpart showed increased PD-L1. Two additional BL cell lines, Jijoye, which is positive for EBNA2 expression expressed PD-L1, while EBNA2-deleted Daudi BL lacked PD-L1 expression (Fig. 1a). The above data suggest that latency III-related viral proteins could influence PD-L1. To extend these observations, we infected two EBV-negative GC DLBCLs, U2932 and SUDHL5 and two EBV-negative BLs, OMA4 and DG75 with a recombinant Akata EBV. The resultant convertants expressed EBNA2 (Fig. 1b, c). PD-L1 expression was strongly upregulated in both DLBCLs (Fig. 1b) and two BLs (Fig. 1c) after in vitro EBV infection.

From data in Fig. 1a (right panel, Daudi−Jijoye comparison), it became clear that EBNA2 might have a critical role in the observed upregulation of PD-L1. To further confirm this, we transfected U2932 DLBCL with an EBNA2 containing expression vector. The transfection and selection conditions of EBNA2 and LMP1 expressing derivatives of U2932 have been previously described in detail elsewhere by us [54]. A strong increase in PD-L1 was observed in EBNA2 transfectants but not in LMP1-transfected U2932 cells (Fig. 1d, left panel). The lack of PD-L1 induction by LMP1 was also confirmed in transfected SUDHL5 DLBCL (supplementary figure 1). PD-L1 induction by EBNA2 was also confirmed by flow cytometry as well in EBNA2-expressing U2932 (supplementary figure 2A). Additionally, in BL41 K3 cells, EBNA2 induction by estradiol treatment was paralleled by an increase in PD-L1 expression (Fig. 1e). PD-L1 upregulation was confirmed by real-time q-PCR in ER/EB 2.5 cell line, which carries estradiol-inducible EBNA2 (supplementary figure 2B).

### Transcription of the PD-L1 targeting miRNA miR-34a is downregulated by EBNA2

**Fig. 2:**
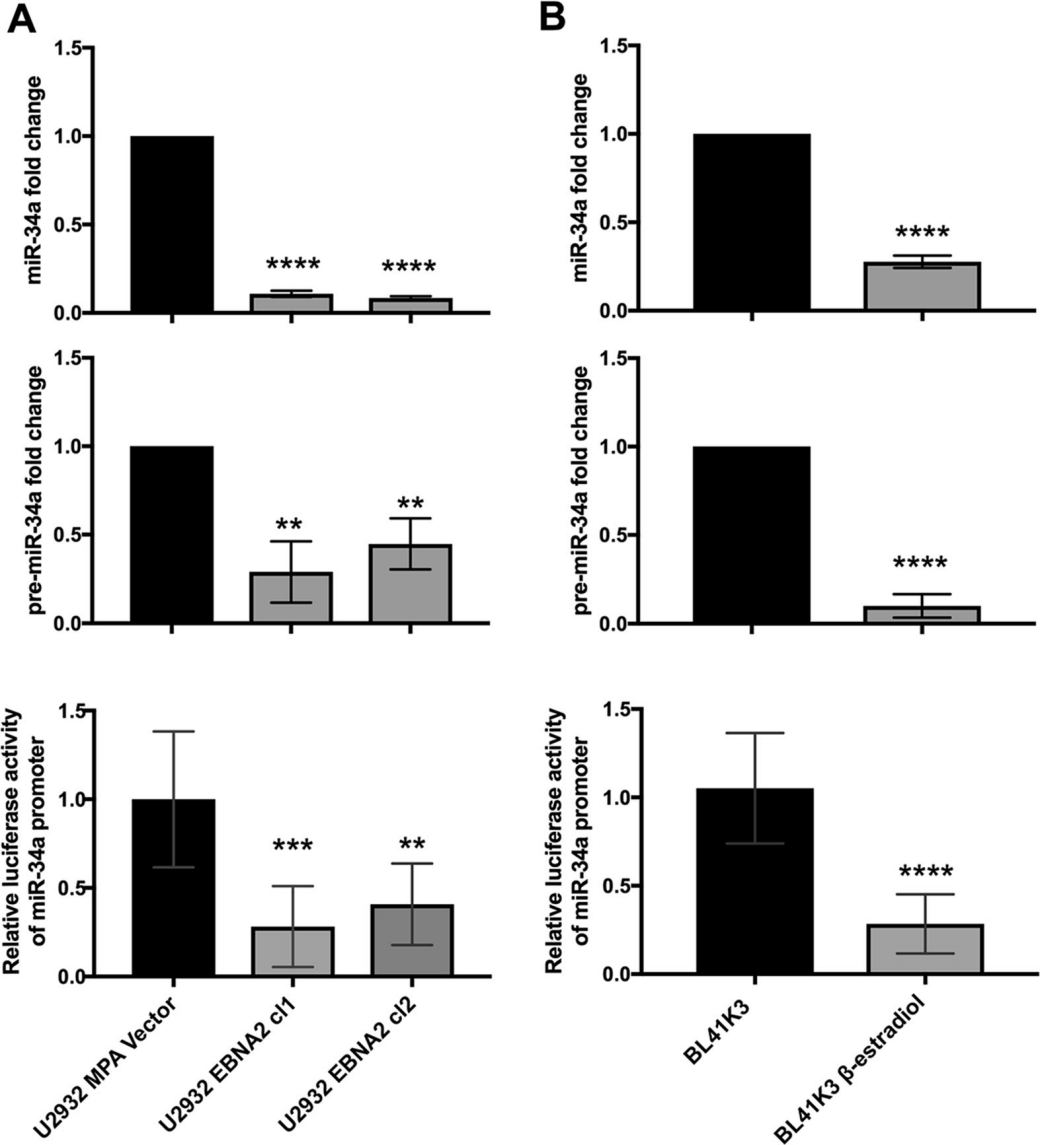
EBNA2 expression decreases miR-34a in transfected B lymphoma cells by affecting its transcription. miR-34a (top panels) and pre-miR-34a expression (middle panels) was verified by real-time qPCR in (**a**) U2932 DLBCL transfected with EBNA2 and (**b**) BL41K3 are transfected with estrogen-inducible EBNA2. miR-34a promoter carrying Luc reporter activity was analyzed in U2932 and BL41 and their EBNA2-expressing derivatives (lower panels). The figure shows standard deviation (SD) of the average of three different experiments. Each experiment was performed in biological triplicates for each sample and repeated three times. *p* values were calculated with unpaired *t* test. In all cell lines miR-34a expression (****) *p* < 0.0001. Pre-miR-34a: U2932 EBNA2 cl-1 (**) *p* = 0.0020 and cl-2 (**) *p* = 0.0027, BL41K3 (****) *p* < 0.0001. MiR-34a promoter: U2932 EBNA2 cl-1 (***) *p* = 0.0002 and cl-2 (**) *p* = 0.0011, BL41K3 (****) *p* < 0.0001

We have previously shown that EBNA2 can profoundly alter cellular miRNA expression profile in U2932 cells [54]. Given the strong increase of PD-L1 expression in EBNA2-transfected BL and DLBCLs and since miR-34a targets PD-L1, we set out to investigate if miR-34a expression is affected in EBNA2-expressing B lymphoma cells. As shown in Fig. 2a, top panel, EBNA2-transfected U2932 cells showed a marked decrease in miR-34a. Similarly, BL41K3 carrying estrogen-inducible EBNA2 showed reduced miR-34a after estrogen treatment (Fig. 2b, top panel). Additionally, both U2932 EBNA2 and BL41K3 cells showed reduced pre-miR-34a expression (Fig. 2, middle panels). To further confirm that the miR-34a decrease is transcriptional, EBNA2-expressing U2932 and BL41 cells were transfected with miR-34a promoter carrying Luc reporters. As seen in Fig. 2 (lower panels), in the presence of EBNA2, the luciferase activity was significantly reduced, confirming that miR-34a is indeed transcriptionally affected by EBNA2.

### Validation of the *PD-L1* 3′UTR as an miR-34a target in U2932 DLBCL

**Fig. 3:**
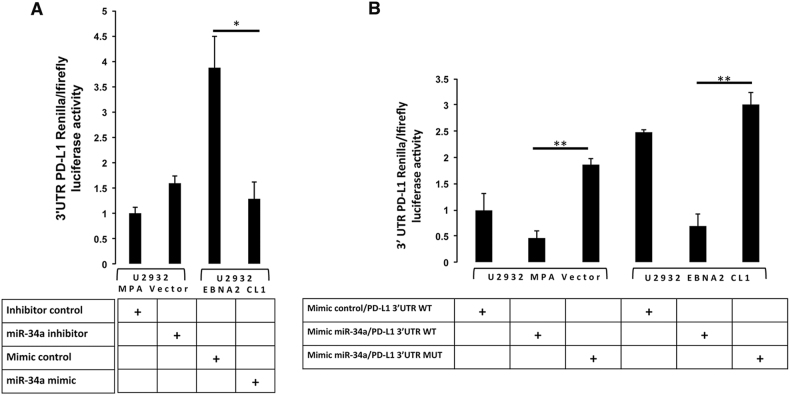
miR-34a targets 3′UTR of PD-L1 in EBNA2-transfected U2932 cells and site-directed mutagenesis of its seed sequence abrogates its binding to PD-L1 3′UTR. **a** Luciferase reporter construct containing wild-type 3′UTR PD-L1 was transfected in presence of either miR-34a inhibitor in MPA vector control transfectants or miR-34a mimic in U2932 EBNA2-expressing clone. Each transfection was performed in triplicate. For U2932 EBNA2 cl-1 is (*) *p* = 0.0172. **b** The specificity of miR-34a binding to its seed sequence in 3′UTR of PD-L1 was confirmed by mutating seed sequence with site-directed mutagenesis. The mimic miR-34a bound to the wild-type 3′UTR of PD-L1 and reduced luc activity. The inhibitory effect of mimic miR-34a was abrogated when its seed sequence in PD-L1 3′UTR was mutated. Each transfection was performed in triplicate. (**) *p* = 0.0026 refers to U2932 MPA Vector and (**) *p* = 0.0021 refers to U2932 EBNA2 cl-1. *p* values were calculated with unpaired *t* test

To investigate the role of miR-34a in the regulation of PD-L1 3′UTR, the complete 3′UTR of *PD-L1* was cloned into a luciferase reporter construct and transfected into U2932 MPA vector and U2932 EBNA2 cells. Subsequently, the miR-34a inhibitors were introduced into the vector alone carrying cells where miR-34a was higher. Instead, miR-34a mimics were transfected into EBNA2-expressing counterparts with low miR-34 expression. Figure 3a shows luciferase activity in controls and in presence of miR-34a inhibitor in U2932 MPA vector or miR-34a mimic in the EBNA2 transfectant. In accordance with miR-34a downregulation in U2932 EBNA2, the luciferase activity was high in these cells. When mimic miR-34a was introduced into EBNA2-expressing cells, the reporter gene activity was significantly reduced (Fig. 3a). To confirm the specificity of miR-34a binding in *PD-L1* 3′UTR, we mutated the miR-34a seed sequence using site-directed mutagenesis. As seen in Fig. 3b, the wild-type 3′UTR reporter activity was high, consistent with low miR-34a in EBNA2-expressing cl-1. When miR-34a mimic was introduced into these cells, the luciferase activity was reduced. In contrast, the mutated seed sequence carrying luciferase reporters were no longer repressed by miR-34a. This not only validated the sequence specificity of the miRNA–mRNA binding but also mapped and confirmed the miR-34a recognition sequence in the *PD-L1* 3′UTR. The absolute expression of miR-34a in U2932 and BL41 parental cell lines and their EBNA2-expressing counterparts in comparison with CD19+ B cells and the Luc activity of wild-type and mutated 3′PD-L1 UTR in both cell lines is shown in supplementary figure 3. In comparison with normal CD19+ B cells, both U2932 and BL41 had higher levels of miR-34a (S Figure 3A). As a consequence, the luciferase activity of the wild-type 3′ PD-L1 UTR construct was repressed, which indicates miR-34a binding to the 3′UTR of PD-L1. In contrast, luciferase activity of the mutated 3′ PD-L1 UTR was not affected by miR-34a. Similarly, in EBNA2-transfected cells, due to lower expression of miR-34a, the Luc activity from both WT and mutated 3′UTR construct was not affected (S Fig. 3B).

### Overexpression of miR-34a in U2932 EBNA2 cells reduces PD-L1

**Fig. 4:**
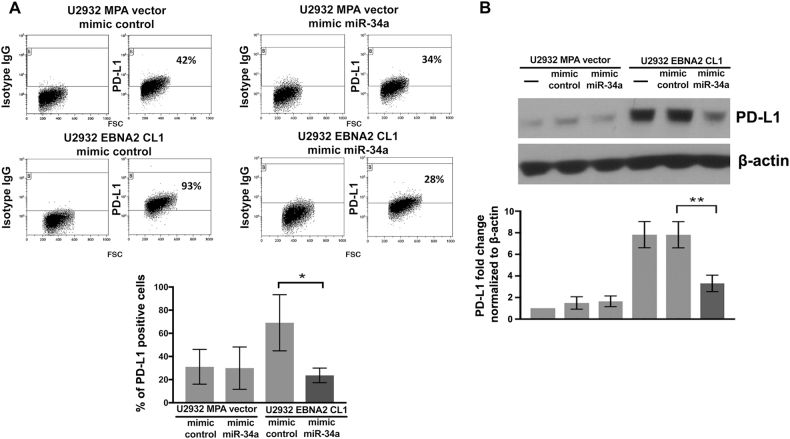
Overexpression of miR-34a in EBNA2-expressing U2932 downregulates PD-L1. **a** miR-34a mimic was transfected into U2932 MPA vector and EBNA2-expressing clone 1. The transfected cells were processed for PD-L1 expression by flow cytometry. The lower panel indicates an average of three flow cytometry experiments and its significance. (*) *p* = 0.0109. **b** The effect of miR-34a reconstitution on PD-L1 expression in the vector control and EBNA2-transfected U2932 cells was also confirmed by immunoblotting. An average of the corresponding densitometric analysis on three such experiments is shown in the lower panel (**) *p* = 0.0055. β-actin served as loading control

**Fig. 5:**
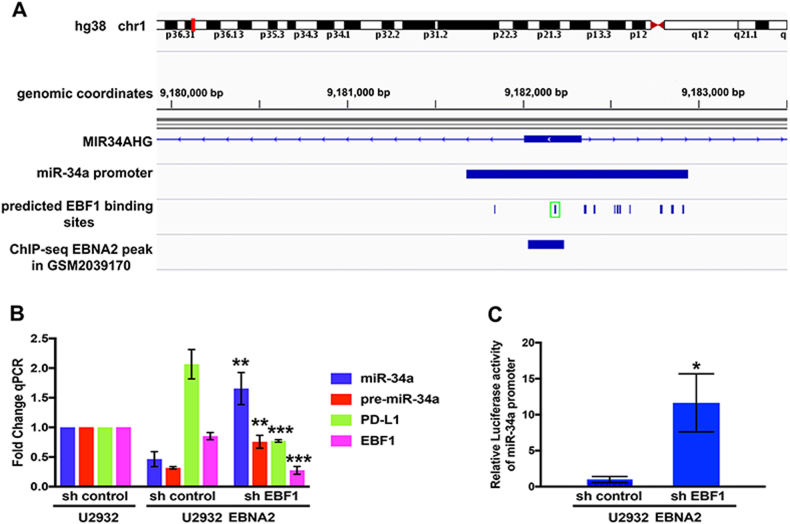
EBNA2 suppresses miR-34a transcription through EBF1. **a** Prediction of EBF1 binding motifs at the miR-34a promoter in human reference genome hg38, coordinates chr1:9181678-9182943, was performed with the JASPAR database and visualized with IGV. EBNA2 peak overlaps with the second of the predicted EBF1 binding sites, highlighted in green square, on the miR-34a promoter in dataset GSM2039170. **b** Knockdown of EBF1 was verified by Q-PCR in the U2932 cell line and U2932 EBNA2 cl1 transduced with pLK0.1 lentiviral vectors which carry shEBF1 and control shRNA. Upon EBF1 depletion (***p = 0.0004) in U2932 EBNA2 cl1, expression of mature miR-34a (**p = 0.0023) and pre-miR-34a (**p = 0.0024) was derepressed and consequently PD-L1 expression decreased (***p = 0.0008). Q-PCRs were performed with three biological and technical triplicates for each sample. **c** MiR-34a promoter activity was enhanced upon EBF1 knockdown in U2932 EBNA2 cl1, after 48 h. Luciferase assay was performed three times and each sample was in triplicate. (*p = 0.0105). Statistical analysis was performed using an unpaired *t* test (Prism-7)

Having established that miR-34a binds to 3′UTR of *PD-L1*, we next investigated if miR-34a overexpression could have a direct effect on PD-L1. For this purpose, we transfected miR-34a mimics in U2932 EBNA2 cl-1. As seen in Supplementary Figure 4, the decrease in Luc activity of the biosensor psicheck-2 construct in the presence of miR-34a mimic clearly suggests its successful delivery and binding to target sequences. To investigate the direct effect of miR-34a on PD-L1, miR-34a-transfected U2932 EBNA2 cl-1 was analyzed for PD-L1. A significant reduction in PD-L1 was observed after overexpression of miR-34a in comparison to the scrambled control (Fig. 4a, b). We further investigated if overexpression of miR-34a in U2932 cells influences p21 and BCL2, previously shown to be regulated by this miRNA [55]. As shown in Suppl Fig. 5A, U2932 EBNA2 cl-1 transfected with miR-34a had an increased p21 but reduced BCL2. Consequently, the number of apoptotic cells was higher in miR-34a-transfected U2932 EBNA2 cl-1 in comparison with the vector transfected cells (S Fig. 5B).

### EBF1 knockdown de-represses miR-34a and downregulates PD-L1 in U2932 EBNA2 cells

Previously reported ChIP-Seq data show that EBNA2 colocalizes with EBF1 at promoter/enhancers of many genes [16]. To identify the molecular mechanism of miR-34a regulation by EBNA2, we analyzed EBNA2 ChIP-Seq datasets from GEO database (accession number: GSM2039170) and found that EBNA2 peaks at the miR-34a promoter. Subsequently, through JASPAR database [56] and visualization through Integrative Genomics Viewer (IGV) [57], using the reference hg38 (human genome38), we found multiple predicted binding sites for EBF1 at the miR-34a promoter, and among them, one consensus EBF1 sequence overlaps with the EBNA2 peak (Fig. 5a, highlighted in green square). Based on this, we reasoned that miR-34a might be regulated by EBNA2 through EBF1. To verify this, the parental U2932 and its EBNA2-expressing derivative line were transduced with lentiviral vectors carrying shEBF1 and shcontrol. As shown in Fig. 5b, upon EBF1 knockdown in U2932 EBNA2 cl1, miR-34a and pre-miR-34a expression is derepressed with a consequential decrease in PD-L1. We further found that miR-34a promoter activity was increased upon EBF1 K.D. (Fig. 5c). These data establish a circuit where EBNA2 might recruit EBF1 to miR-34a promoter to downregulate its expression and consequently upregulate PD-L1.

### Suppression of T-cell activation by EBNA2 and increased immunogenicity after miR-34a overexpression as measured in MLR and 3D biomimetic microfluidic platforms

**Fig. 6:**
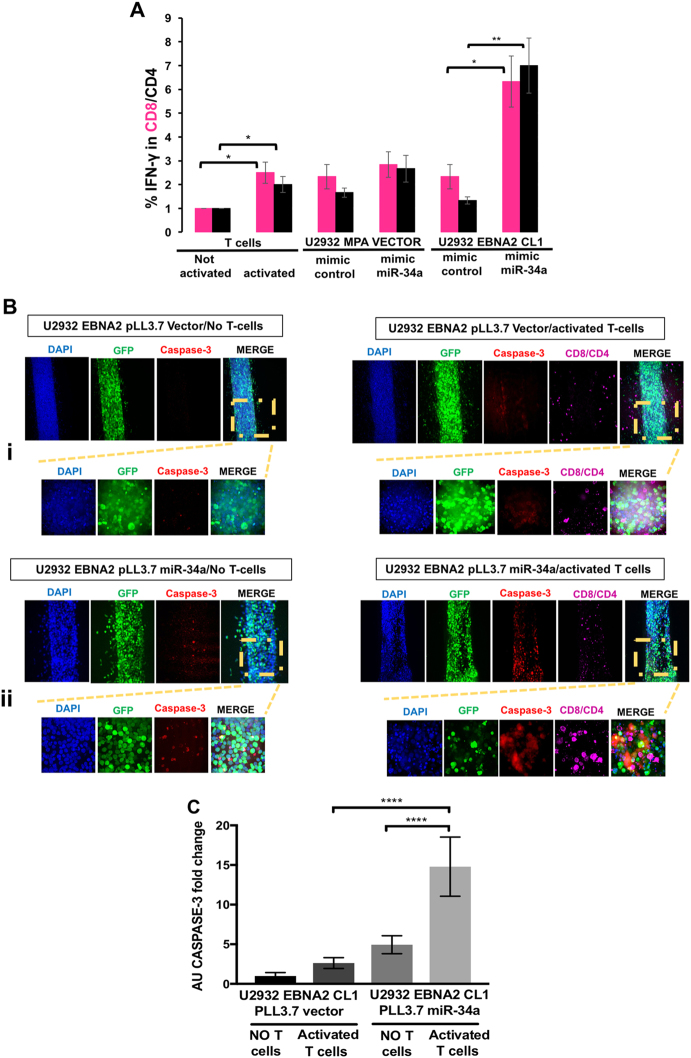
miR-34a relieves suppression of immunogenicity induced by EBNA2 as measured in MLR and 3D biomimetic microfluidic coculture devices. **a** T cells were activated in plates coated with anti-CD3/anti-CD28 antibodies for 72 h. Irradiated targets U2932 MPA vector and U2932 EBNA2 cl-1 were cocultivated with activated T cells (effector). The effector-target ratio was 1:10. The target cells were transfected with mimic control or miR-34a mimic 24 h prior to cocultivation with the effector cells. The coculture was carried out for 48 h and the cells were stained for CD4/CD8 and IFN-γ and processed for flow cytometry. Data are expressed as mean ± SD. The *p* values for T-cell activation without stimulators are (*) *p* ≤ 0.05 for CD8 and CD4. In MLR with U2932 stimulators, the statistical significance is (*) *p* = 0.028 for CD8 and (**) *p* = 0.0081 for CD4. Three different experiments were performed with PBMCs isolated from three different donors. **b** Three-dimensional biomimetic microfluidic coculture devices: Four million/ml U2932 EBNA2 cells tranduced with lentiviral vector controls were introduced into the microfluidic devices. In coculture experiments (right panel in **b**), devices were first seeded with U2932 EBNA2 cells and were incubated for 24 h at 37 °C, followed by activated T cells seeding. Immunostaining was performed after 48 h of cocultivation. **bi**: Representative confocal images of U2932 EBNA2 cl-1 transduced with GFP-lentiviral vector control in the absence or presence of activated T cells; **bii** Four million/ml U2932 EBNA2 cl 1, tranduced with miR-34a lentivirus, were introduced in the collagen/fibronectin devices either alone (left panel) or cocultivated with previously activated T cells (right panel). The cocultivation of target, miR-34a transduced U2932 EBNA2 cells with activated T cells, was carried out for 48 h before immunostaining. miR-34a containing lentivirus-transduced U2932 EBNA2 cl-1 cells were stained with anti-GFP antibody (green), activated CD8/CD4 T cells were stained with anti-CD8 and anti-CD4 (magenta), apoptotic U2932 EBNA2 cl-1 cells were visualized with anti-caspase-3 antibody (red), and nuclei were counterstained with DAPI (blue). The overlap of miR-34a transduced U2932 EBNA2 cells (GFP positive) and caspase-3 (red) indicates tumor cell death (merged images, yellow). Scale bar = 100 μm, magnification of the insets = scale bar 20 μm. **c** Arbitrary Caspase-3 units were calculated for each experimental condition. Statistically significant caspase-3 positive cells were observed only in miR-34a tranduced EBNA2 expressors; Data expressed as mean ± SEM; (****) *p* < 0.0001. *N* = *4* fields (3–4 devices for each experimental condition). Shown is one representative experiment out of four performed. Statistical analysis was performed with Prism-7 software using unpaired *T* test

In order to understand the immunological relevance of PD-L1 upregulation and miR-34a downregulation by EBNA2, we first employed an MLR assay. After 3 days of PBMC activation on CD3/CD28-coated wells, the irradiated stimulator U2932 MPA vector, U2932 EBNA2 cl-1 and either their mimic control or miR-34a-transfected derivatives were added in an MLR. Successful miR-34a delivery in stimulator cells and its binding to specific target sequence was confirmed using the psicheck-2 biosensor reporter assay (Suppl. Figure 6A). Effector T-cell activation was confirmed by a strong increase in PD-1 expression in two donors (Suppl. Figure 6B). The activated T-cell state was corroborated by increased IFN-γ production (Fig. 6a). Importantly, U2932 EBNA2 cl-1 boosted IFN-γ production, by both CD8 and CD4 T cells, only when miR-34a was overexpressed (Fig. 6a). These data suggest that the increase in PD-L1 by EBNA2 may have a negative effect on T-cell activation and reconstitution of miR-34a restores immunogenicity of EBNA2 transfectants.

We next investigated how miR-34a might reverse the poor immunogenicity of EBNA2-transfected high PD-L1-expressing U2932 cells. The schematic design of the 3D microfluidic chip-based coculture system is shown in Suppl. Figure 7. The effector T-cell activation was confirmed by increased IFN-γ (Suppl. Figure 8A). Stimulator U2932 EBNA2 cells tranduced either with lentiviral vectors carrying miR-34a or vector control were introduced into microfluidic devices. The expression of miR-34a in lentivirus-transduced U2932 EBNA2 cells was checked by real-time qPCR (Suppl. Figure 8B) and the consequent PD-L1 decrease was verified by flow cytometry (Suppl. Figure 8C). Figure 6Bi shows the device with empty lentiviral vector-transduced U2932 EBNA2 expressors either in the presence or absence of T cells. No significant change in caspase-3 expression was observed. In contrast, as seen in Figure 6bii, when miR-34a containing lentivirus was transduced into EBNA2 U2932 clone, there was a marginal induction of caspase-3 in the absence of T cells, most probably due to apoptosis induced by miR-34a expression. In contrast, overexpression of miR-34a in EBNA2-expressing U2932, in the presence of CD4/CD8 cells, induced significant tumor cell death, as indicated by increased caspase-3 expression (Fig. 6bii, c). Overall, these data suggest that reconstitution of miR-34a in EBNA2-expressing U2932 makes them more immunogenic.

### PD-L1 and EBV correlation in clinical DLBCL samples

**Fig. 7:**
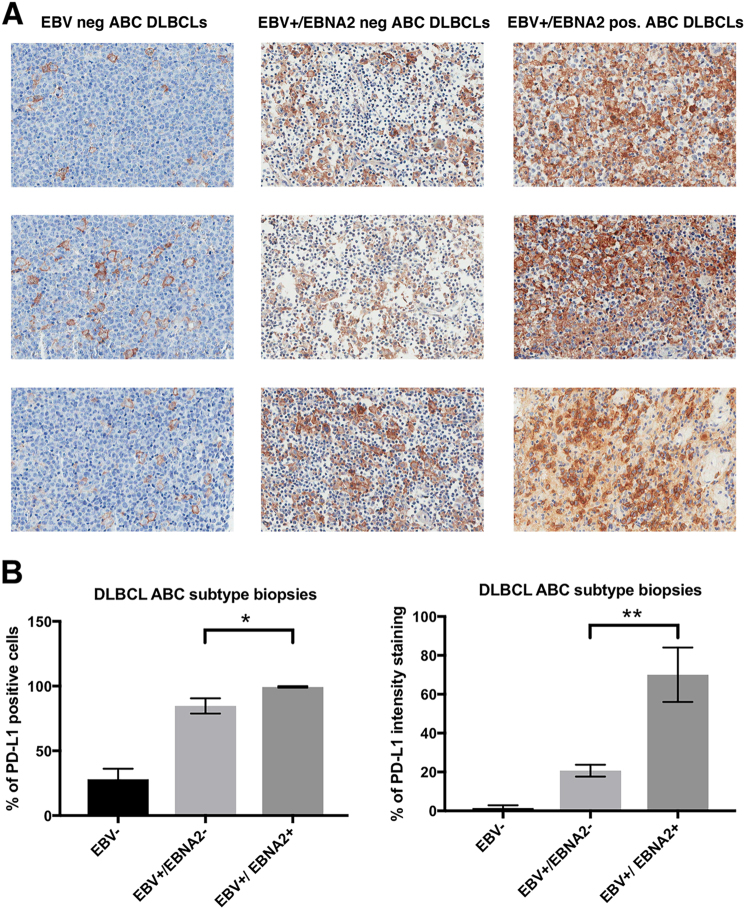
PD-L1 expression in DLBCL clinical tissues. **a** Three non-GC DLBCL patient samples representing the three ABC DLBCL categories, out of a total of 21, stained for PD-L1 are shown. Paraffin sections were immunostained for PDL1 using an automated immunostainer (DAKO, Glostrup, Denmark). As control for PDL-1 immunostaining, sections from paraffin-embedded human lung carcinoma were used. **b** The stained tissue sections were digitalized at a ×40 magnification using Aperio Scan Scope. The percentage positivity was calculated by counting positive cells in three squared areas measuring 50,000 μm^2^ from each clinical sample in (**a**) above. The number of positive cells was determined using Aperio software IHC Membrane v1. This algorithm detects membrane staining for individual tumor cell in the selected regions and quantifies the intensity and completeness of the membrane staining. Unpaired *t* test was applied to demonstrate that differences in % total PD-L1-positive cells and % cells with strong staining intensity were statistically significant, (*) *p* = 0.0125, (**) *p* = 0.0040

In a cohort of 27 cases of DLBCLs, we investigated how EBV and EBNA2 expression is correlated with increase in PD-L1 expression. According to the Hans Algorithm, 21 cases were classified as non-GC type and 6 cases as GC type. Figure 7a shows PD-L1 expression in three non-GC DLBCLs representing each category namely, EBV negative, EBV+/EBNA2−, and EBV+/EBNA2+ samples. PDL-1 expression was detected at the cell membrane level, in the cytoplasm or as dots in the Golgi area of the neoplastic cells. For quantitative estimation of PD-L1 expression and staining intensity, Aperio Imagescope analysis was employed. The stained tissue sections were digitalized at a ×40 magnification using Aperio Scan Scope. The percentage positivity was calculated by counting positive cells in three squared areas measuring 50,000 μm^2^ from each clinical sample. In the same areas the number of the positive cells was determined using the Aperio software IHC Membrane v1. The IHC Membrane Image Analysis algorithm detects membrane staining for individual tumor cells in the selected regions and quantifies the intensity and completeness of the membrane staining. Figure 7b (upper panel) shows that there was a slight and statistically significant overall increase in PD-L1-positive cells in EBNA2-positive cases. Notably, as shown in Fig. 7b, in all EBNA2+ samples analyzed, the number of cells with high staining intensity (+2, +3) as measured by Imagescope algorithm was significantly higher in EBNA2+ ABC DLBCLs in comparison with EBNA2− cases (Supp table 1).

We also analyzed PD-1 expression and found that it is generally expressed by infiltrating cells like T lymphocytes (TILs) and macrophages and not by the neoplastic cells. There was no correlation between the number of PD-1-positive infiltrating cells and PDL-1 expression by neoplastic cells (not shown). Suppl. Table 1 describes the details of the clinical samples.

## Discussion

Viruses, being obligate parasites, are under constant pressure to survive in the face of strong host immune responses. To maintain a replicative advantage, they use multiple strategies to make themselves immunologically invisible. This includes downregulation of HLA class I, class II molecules, interference with peptide transport mechanisms, inhibition of proteolysis etc. [58]. In this regard, akin to many other viruses, EBV also employs several mechanisms to circumvent immune eradication to establish latency. EBV-positive DLBCLs are high PD-L1 expressors and this is confirmed here [31]. However, which virally encoded proteins could be delegated with this task and how do they achieve it has not been fully explored. To the best of our knowledge, this is the first report of how EBV, through its most critical transformation-associated protein, EBNA2, affects PD-L1 expression both in DLBCLs and BLs, by downregulating miR-34a through recruitment of EBF1 to its promoter. In the first ever use of a microfluidic chip for EBV-associated lymphoma growth in 3D, we further show that EBNA2-expressing DLBCLs are less immunogenic. Reconstitution of miR-34a in U2932 EBNA2 cells increased their immunogenicity as seen by IFN-γ production in MLRs and increased apoptosis as measured by caspase-3 expression in tumor T-cell 3D cocultures.

Most EBV-positive DLBCLs are non-GC type and high PD-L1 expressors. But it is not known if EBV directly infects a non-GC DLBCL or whether it actually could turn a GC DLBCL into a relatively activated DLBCL. Our data showing strong upregulation of PD-L1 in two, in vitro infected GC DLBCLs suggest that EBV indeed has the ability to turn a GC-derived DLBCL into at least a partially activated one. It is important to clarify here that U2932, often described in the literature as ABC type, is a high BCL6, a hallmark of GC phenotype, expressing cell line [14]. Furthermore, a recent detailed classification study suggests that BCL6 is critical marker of GC DLBCL category [59]. Additionally, most ABC DLBCLs express PD-L1. In contrast GC DLBCLs are often PD-L1 negative. U2932 DLBCL is indeed PD-L1 negative. Based on this, we consider U2932 more as an intermediate phenotype DLBCL. Patients with non-GC or activated DLBCLs have both poor prognosis and overall survival rate [31–34]. Results from our clinical DLBCL samples suggest that EBV-positive non-GC DLBCLs have slightly higher PD-L1 expression than those non-GC DLBCLs without the virus. A quantitative IHC image algorithm analysis on digitalized slides revealed that in EBNA2-positive ABC DLBCL samples PD-L1 expression and the staining intensity was higher. Clearly, the effect of EBNA2 alone on PD-L1 would be impossible to determine in clinical samples because EBNA2 alone latency does not occur in any tumor associated with EBV. But, notwithstanding the small cohort, the data from clinical samples confirm the in vitro data. Overall, we suggest that the effect of EBNA2 on PD-L1 in clinical samples will have to be tested in a larger cohort. However, the results are consistent with the suggestion that EBNA2-positive lymphomas may have a better therapeutic outcome with IC blockers.

MiR-34a belongs to the group of tumor suppressor miRNAs and accordingly, it is frequently downregulated in a wide variety of cancers [60]. In keeping with this, its expression is often reduced in ABC type of DLBCL cell lines and tumor tissues [61]. Overall survival of those patients with low miR-34a is poorer and overexpression of miR-34a in ABC DLBCL lines makes them responsive to doxorubicin treatment [61]. Our observations that EBNA2 downregulates miR-34a are consistent with the reported lower expression of miR-34a in ABC DLBCLs and doxorubicin resistance [61]. Indeed, in Lat III ABC DLBCLs, EBNA2 might contribute to chemoresistance and poor prognosis by downregulating miR-34a. Additionally, Craig et al. have shown that intravenous miR-34a treatment of mice with U2932 DLBCL xenografts suppresses tumor growth, thus underpinning its therapeutic utility [62]. Among its noted targets is the oncogene FOXP1 [63]. Interestingly, in AML, miR-34a targets PD-L1 [29, 64]. We now show that EBV, through its growth transformation-associated protein EBNA2, increases PD-L1 by downregulating miR-34a. Furthermore, in the presence of EBNA2, pre-miR-34a and miR-34a promoter activity is reduced and this suggests that EBNA2 affects miR-34a transcription.

We found that miR-34a downregulation by EBNA2 likely involves recruitment of EBF1 at the miR-34a promoter. Glaser et al. have recently shown that EBF1 interacts with the N-terminal portion of EBNA2 in a B-cell specific manner and this interaction promotes EBNA2 access to chromatin, without involving RBPJk, a known EBNA2-DNA anchor [19]. Our analysis of EBNA2 ChIP-Seq datasets from GEO database (accession number: GSM2039170) revealed that EBNA2 peaks at the miR-34a promoter. Furthermore, the data showing the importance of EBF1 in miR-34a regulation by EBNA2 is consistent with previous suggestion that EBNA2 and EBF1 are colocalized at EBNA2 peaks [19]. Recently it was also shown that Ten-Eleven translocation 2 (TET2) is highly expressed in latency III (EBNA2+) BLs and ABC DLBCLs [65]. Interestingly, EBNA2 colocalizes with both EBF1 and TET2 [16, 66]. From our data, the role of EBF1 in negative regulation of miR-34a is evident but the possibility that EBNA2 could influence PD-L1 by affecting TET2 needs further investigation. Overall, our data support the notion that EBNA2/EBF1 involvement in miR-34a regulation can be therapeutically harnessed for DLBCL and particularly for the drug resistant cases.

As mentioned earlier, EBNA2 is the main driver of B-cell transformation induced by EBV. To this end, it is noteworthy that c-MYC is directly upregulated by EBNA2 [12]. Additionally, EBNA2 is also a functional homolog of activated Notch [15]. Both c-MYC and activated Notch are known for their oncogenic properties. Most interestingly, both these proteins are miR-34a targets [67, 68]. Based on our data, we surmise that EBNA2 may not only be the functional homolog of Notch but indeed it may help keep Notch expression up through downregulation of miR-34a. Casey et al. have recently shown that c-MYC can induce PD-L1 expression [69]. Further studies will be required to understand if EBNA2 by downregulating miR-34a increases c-MYC, which in turn may upregulate PD-L1. At present, it is not known if activated Notch genes like c-MYC have any effect on PD-L1 expression. Based on our data, this exciting possibility needs further investigation.

Increased tumorigenicity is often combined with poor immunogenicity in cancer. Thus, the double-edged sword-like function of EBNA2 to downregulate miR-34a through EBF1 and consequently upregulate PD-L1 adds to the long list of its oncogenic attributions. To argue against its relevance, because EBNA2 expression is a rarity in lymphomas, would be fallacious, particularly, if wider implications of our findings are considered. EBV-induced immunoblastomas of immunocompromised patients, such as in AIDS and transplant, are EBNA2 expressors. A significant proportion of cases within EBV-positive ABC DLBCLs are also EBNA2 positive. The viral gene expression pattern in these tumors resembles that of in vitro transformed LCLs and cellular proliferation in both these cell types is indeed EBNA2 driven. Clearly, in patients with compromised T-cell immune responses, therapeutic approaches like inactivation of EBNA2 by Crispr-Cas9 gene editing and/or therapeutic introduction of miR-34a mimics will have to be considered.

The 3D biomimetic microfluidic devices, described here for the first time to test immunogenicity of lymphoma cells, provide a quick and economically viable alternative to a more expensive and cumbersome, humanized mouse-based approaches for human tropic viruses like EBV. In addition, these devices might also prove useful in testing the efficacy of combinatorial immunotherapy agents, in lieu of humanized mice.

In conclusion, the identification of EBNA2 as a lead player in tampering with immunogenicity of EBV-infected cells by altering PD-L1 and miR-34a opens up several new RNA-aided immunotherapy avenues to explore. We propose a combinatorial delivery of antibodies and miR-34a to silence PD-L1 both from within and without the cell to maximize chances of a successful and potent therapy to benefit immunocompetent patients with EBV-associated cancers, but such an approach might have wider implications for other cancers as well.

## Electronic supplementary material

S material and methods

S figure legends

S figure 1

S figure 2

S figure 3

S figure 4

S figure 5

S figure 6

S figure 7

S figure 8

S Table 1
