# Supplementary material for: Epstein−Barr virus-encoded EBNA2 alters immune checkpoint PD-L1 expression by downregulating miR-34a in B-cell lymphomas
Source: Leukemia. 2018 Jun 26;33(1):132–47. doi: 10.1038/s41375-018-0178-x (PMC6327052; doi:10.1038/s41375-018-0178-x)
Supplement: Supplementary file 1 — S material and methods [file 41375_2018_178_MOESM1_ESM.docx]

**Supplementary material and methods:**

**Immunoblotting:**

The working conditions of all antibodies were those indicated by the respective providers. The chemiluminescence kit (Amersham) was employed to visualize the proteins. Three million cells were lysed in 100µl of lysis buffer (cell signaling) and the protein concentration was estimated by Bradford assay. Protein extracts equivalent to 30µg were separated on discontinuous SDS-polyacrylamide gels and transferred to Protran filters (Amersham).

**Real time qPCR**:

For real time qPCR, to each DNAse treated RNA sample, 33 ng random hexamers, 2 µl of 10mM dNTPs were added and incubated at 70° C for 5 min. The following reaction components were added: 12 µl of 5x 1^st^ strand buffer (Superscript III reverse transcriptase, Invitrogen), 1,44 µl RNAsin (Promega) and 17,5 µl of DEPC water. The final volume of 60 μl was split into 2x30 µl aliquots. To one aliquot, 1 µl (200 units) of superscript III reverse Transcriptase (Invitrogen) was added and to the other, 1 µl of DEPC water to control for genomic DNA contamination. The samples were then incubated for 10 minutes at 25° C, 50 min at 37° C and 15 min at 70° C. Subsequently, each cDNA sample was split into three and used for qRT-PCR. The mature miR-34a and the housekeeping RNU6B were amplified with the miScript primer assay (QIAGEN). Pre-miR-34a expression was evaluated by employing miScript Precursor assay and QuantiTect primer assay S18 (QIAGEN) used as a housekeeping gene. For miR-34a and RNU6B amplification, the Q-PCR reaction was performed with miScript SYBR Green PCR Kit on a real time Roche LightCycler PCR system. Pre-miR-34a and S18 amplification were performed with LightCycler 480 SYBR green master mix I (Roche). The relative amount of pre-miR-34a was calculated by comparative Ct (threshold cycle) method by subtracting the average Ct value for the housekeeping genes from the average Ct value for each miR and pre-miR (ΔCt). Next, the ΔΔCt values were calculated by subtracting the ΔCt value of the common calibrator (U2932 parental DLBCL cells) from the ΔCt of each sample. Finally, the relative expression was defined as 2^-ΔΔCt^ . The range of expression levels was determined by calculating the standard deviation of the 2^-ΔΔCt^ of three independent experiments, each starting from a new RNA extraction.

**Flow Cytometry Analysis:**

To characterize PD-L1 expression on the cell surface of U2932 cells and their EBNA2-transfected derivatives, we conducted ﬂow cytometry analysis by using a monoclonal Antibody to PD-L1 from eBioscience Inc. (San Diego, CA, USA) with its matched isotype control IgG2a as we did in Fixable viability dye was also included in the analysis to exclude dead cells. U2932 were ﬁxed with 2% paraformaldehyde before analysis on a FACSCanto (BD Pharmingen). A total of 50.000 live cells were analyzed per samples. Data analysis was performed by using FlowJo software (TreeStar Inc., Ashland, OR, USA).

**Apoptosis assay by annexin V:**

To study whether miR-34a has any effect on apoptosis, 0.5x10^6^ U2932 MPA vector and U2932 EBNA2 expressing cells, were transiently transfected with mimic miR-34a or mimic control. The cells were harvested and washed in PBS after 24h and 48h post transfection and stained for apoptosis according to APC Annexin V Apoptosis Detection with PI Kit instructions (BioLegend). Detection of the percentage of early apoptotic (only Annexin V) late apoptotic (both Annexin V/PI) and necrotic cells (only PI) was done at 24 and 48h post transfection, by using Gallios flow analyzer (Beckman Coulter) and the data were analyzed with Kaluza for Gallios Software.

**Lentivirus production:**

Supernatants containing the lentivirus were collected at 24h, 48h and 72h post infection and filtered with 0.45µm sterile filters (Millipore) and concentrated 30x with Amicon Ultra-15, 100k (Millipore). Lentivirus titers were estimated with Lenti-XGoStix (Clontech). Lentivirus was then mixed with 8 µg/ml polybrene (Millipore) and added to confluent U2932 MPA vector and U2932 EBNA2 cells, placed in triplicates in a 24 well plate and spinoculated for 1 hour at room temperature. After 72h, transduced cells expressing GFP underwent flow cytometric sorting and used for further analysis.

**Immunohistochemistry of DLBCL clinical samples:**

In situ hybridization for EBV-encoded RNA (EBER) was performed on paraffin sections using EBV (EBER) PNA probe/Fluorescein and FITC/HRP (DAKO, Glostrup, Denmark). The Hans algorithm (CD10, Bcl-6, MUM-1) was used to classify germinal center (GC) type or non-GC type DLBCL.

Immunostaining results were recorded as positive and negative cases. In positive cases the staining intensity was arbitrarily measured as: +1 weak, +2 intermediate, +3 strong. Following antibodies were used: PDL-1 (E1L3N) (Cell Signaling), PD-1 (DAKO, Glostrup, Denmark), LMP-1, MUM-1, CD10, Bcl-6 (DAKO, Glostrup, Denmark).
