# Supplementary material for: Epstein−Barr virus-encoded EBNA2 alters immune checkpoint PD-L1 expression by downregulating miR-34a in B-cell lymphomas
Source: Leukemia. 2018 Jun 26;33(1):132–47. doi: 10.1038/s41375-018-0178-x (PMC6327052; doi:10.1038/s41375-018-0178-x)
Supplement: Supplementary file 2 — S figure legends [file 41375_2018_178_MOESM2_ESM.docx]

**Supplementary Figure legends:**

**S Figure 1: Expression of PD-L1 in LMP-1 transfected clones, in SUDHL5**

High LMP1 expression does not increase PD-L1 in SUDHL5 LMP1 transfected cells.

**S Figure 2: A: Detection of PD-L1 by flow cytometry in U2932 and its EBNA2 expressing derivatives and B: PD-L1, miR-34a and pre-miR-34a by real-time qPCR in hormone inducible EBNA2 transfected ER/EB 2.5 cells.**

A: Flow cytometry: One representative experiment out of five. MFI: Mean fluorescence intensity. B: PD-L1, pre-miR-34a and miR-34a expression was analyzed by real time q-PCR in estradiol treated ER/EB2.5 cells. *** p=0.0002 and **** p<0.0001. q-PCR was repeated three times.

**S Figure 3: Expression of miR-34a in U2932 and BL41 cells comparing with normal CD19^+^ B-cells**

**A:** MiR-34a is highly expressed in U2932 and BL41 cell lines, while miR-34a expression is reduced in EBNA2 transfected cells, comparing with CD19+ B-cells from healthy donors. mean±SD of three different experiments.

**B:** Wild type (WT) 3’UTR PD-L1 luciferase activity increases in the presence of EBNA2 in both cell lines, U2932 and BL41, confirming the lack of endogenous miR-34a binding to the seed sequence of the 3’UTR of PD-L1. Contrarily, endogenous miR-34a in both parental cell lines reduced the luciferase activity of WT 3’UTR of PD-L1. The luciferase activity of 3’UTR of PD-L1 mutated in miR-34a seed sequence was not affected. The experiments were performed three times and in triplicates. The results are shown as the mean±SD.

**S Figure 4: Detection of miR-34a activity in U2932 MPA vector and U2932 EBNA2 cells co-transfected with miR-34a mimic or mimic control**

U2932 MPA vector and U2932 EBNA2 cl-1 were co-transfected with miR-34a and miR-34a mismatch biosensor in combination with mimic control or miR-34a mimic. At 48 h post-transfection cells were analyzed for miR-34a luciferase activity. A strong reduction of luc activity was observed in both U2932 MPA vector and EBNA2 cl-1 co-transfected with mimic-miR-34a and miR-34a biosensor. This confirms both specificity and successful delivery of miR-34a in these cells, which were used for further experiments (flow cytometry, Western blot and apoptosis assay). Each sample was transfected in triplicate and the experiment was repeated at least three times. Error bars represent SEM; (***) p=0.0003 for the parental cell line U2932 MPA Vector and (***) p=0.0001 for U2932 EBNA2 cl-1.

**S Figure 5: miR-34a over-expression in EBNA2 transfected U2932 cells affects expression of target genes and apoptosis**

**A**: P21 is induced and BCL-2 is downregulated by miR-34a. The expression of these two proteins was tested in miR-34a transfected U2932 and EBNA2 expressing clone. Indeed, p21 was induced and bcl2 was downregulated at 48h post-transfection of miR-34a. **B**: Either mimic control or miR-34a mimic transfected U2932 MPA vector and U2932 EBNA2, were stained for apoptosis according to APC Annexin V Apoptosis Detection kit. The detection of the percentage of early apoptotic (only Annexin V) late apoptotic (both Annexin V/PI) and necrotic cells (only PI) was done at 24 and 48h post transfection, by using Gallios flow analyzer (Beckman Coulter) and the data were analyzed with Kaluza for Gallios Software. One representative experiment of four is shown.

**S figure 6: Verification of miR-34a transfection in stimulator cells and activation of effector T cells used in standard MLR**

**A**: miR-34a biosensor was transfected together with either mimic control or miR-34 mimic. Reduction in luciferase activity in U2932 MPA vector and U2932 EBNA2 cells confirms successful expression of miR-34a. Cells were transfected in triplicates. Error bars represent SEM;

(****) p<0.0001. **B**: The activation of effector T cells within a PBMC population isolated from two different healthy donors, donor A and donor B, was verified by expression of PD-1 on both CD4+ and CD8+ T cells by flow cytometry.

**S Figure 7: Schematic representation of a microfluidic platform for 3D mixed lymphocyte culture**

The 3D model devices were seeded with U2932 EBNA2 cells transduced with either miR-34a containing PLL3.7 lentivirus or the corresponding vector control, carrying the GFP marker. 24 hours later, activated T cells were added and after another 48 hours, the devices were processed for caspase-3 staining. The cross section indicates how the cells were placed inside the devices.

**S Figure 8: Immunogenicity assays on microfluidic chips: Verification of T cell activation, expression of miR-34a in lentivirus transduced target U2932 EBNA2 cells and consequent downregulation of PD-L1**

**A**: Before loading on the microfluidic chips for 3D MLR, the activation of T cells was confirmed by analyzing IFN-γ by flow cytometry **B**: miR-34a expression was verified by qPCR in miR-34a lentivirus and vector control virus transduced U2932 EBNA2 cl-1 cells. **C**: Downregulation of PD-L1 in U2932 EBNA2 cells after miR-34a transduction was tested by flow cytometry.
