# Supplementary figures and images for: Epstein−Barr virus-encoded EBNA2 alters immune checkpoint PD-L1 expression by downregulating miR-34a in B-cell lymphomas
Source: Leukemia. 2018 Jun 26;33(1):132–47. doi: 10.1038/s41375-018-0178-x (PMC6327052; doi:10.1038/s41375-018-0178-x)

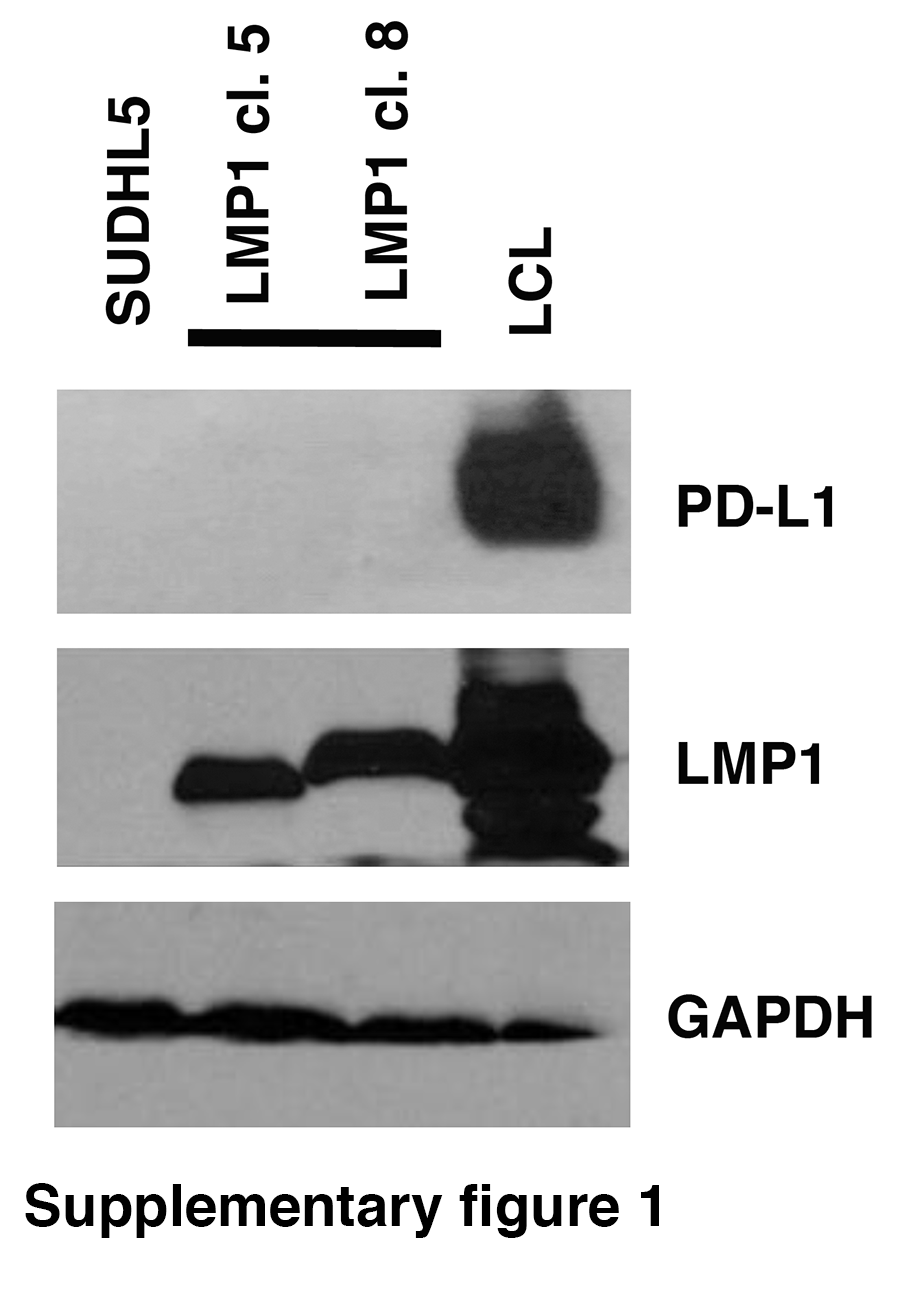

Supplement: Supplementary file 3 — S figure 1 [file 41375_2018_178_MOESM3_ESM.tif]

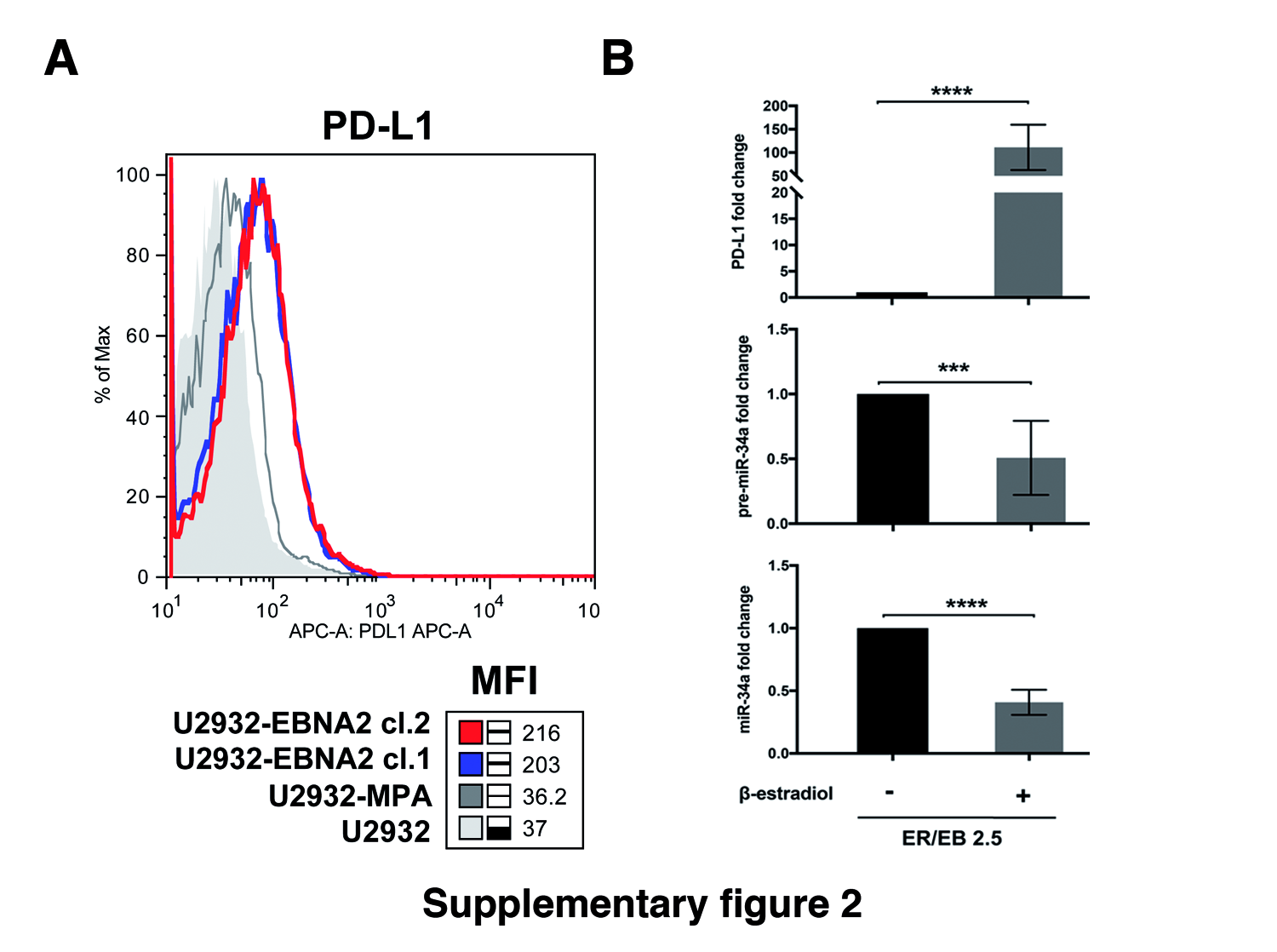

Supplement: Supplementary file 4 — S figure 2 [file 41375_2018_178_MOESM4_ESM.tif]

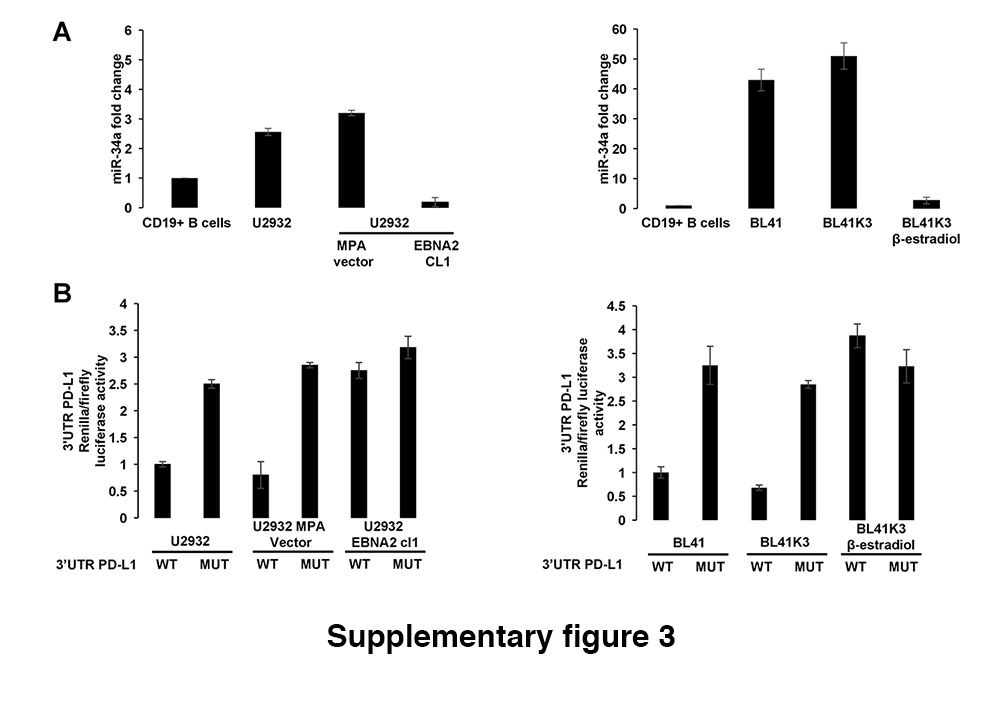

Supplement: Supplementary file 5 — S figure 3 [file 41375_2018_178_MOESM5_ESM.tif]

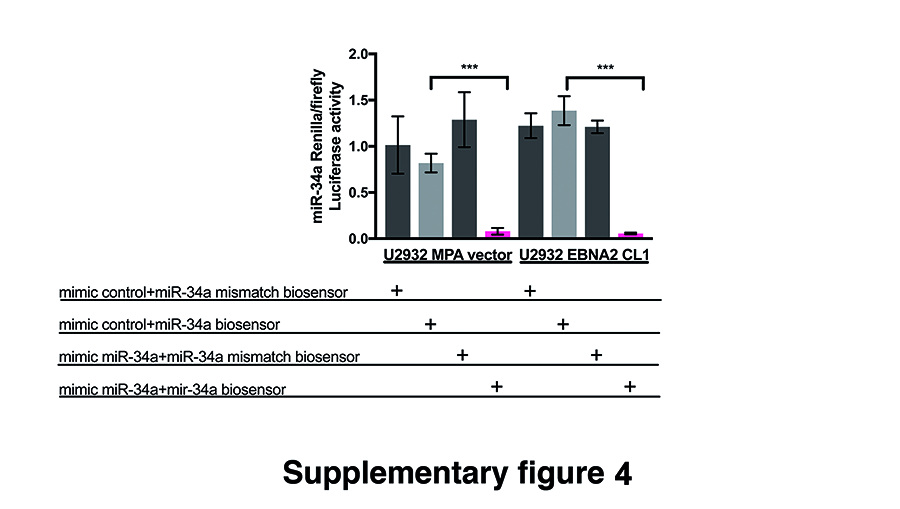

Supplement: Supplementary file 6 — S figure 4 [file 41375_2018_178_MOESM6_ESM.tif]

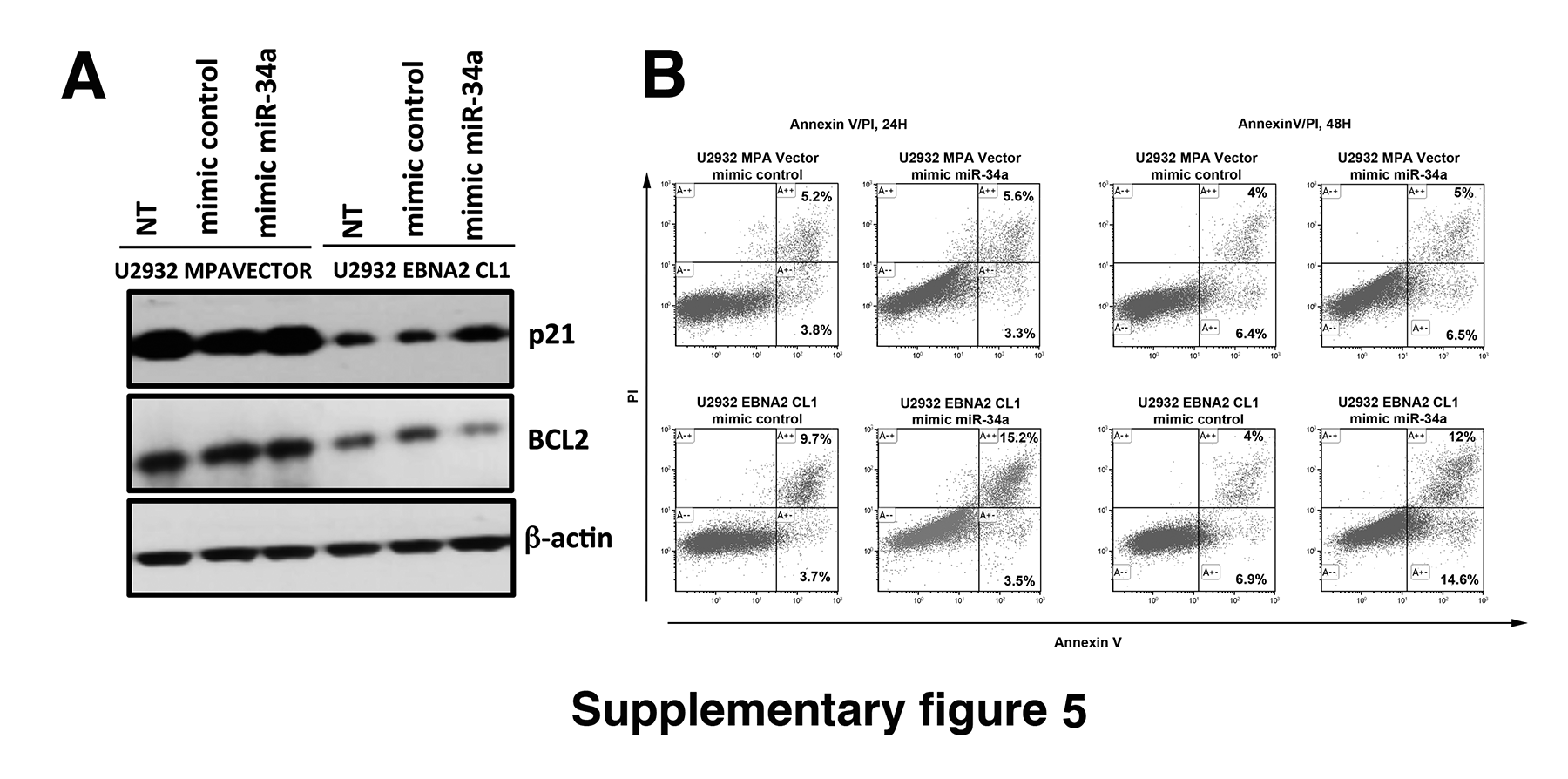

Supplement: Supplementary file 7 — S figure 5 [file 41375_2018_178_MOESM7_ESM.tif]

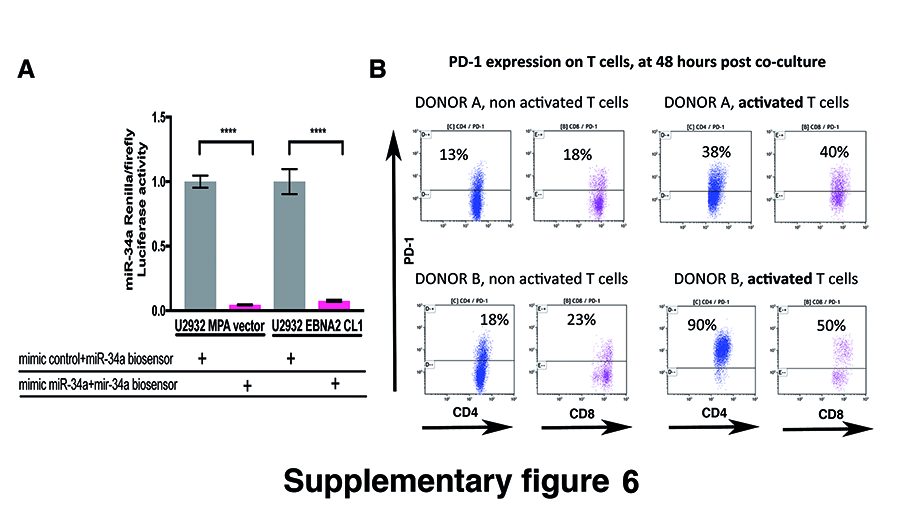

Supplement: Supplementary file 8 — S figure 6 [file 41375_2018_178_MOESM8_ESM.tif]

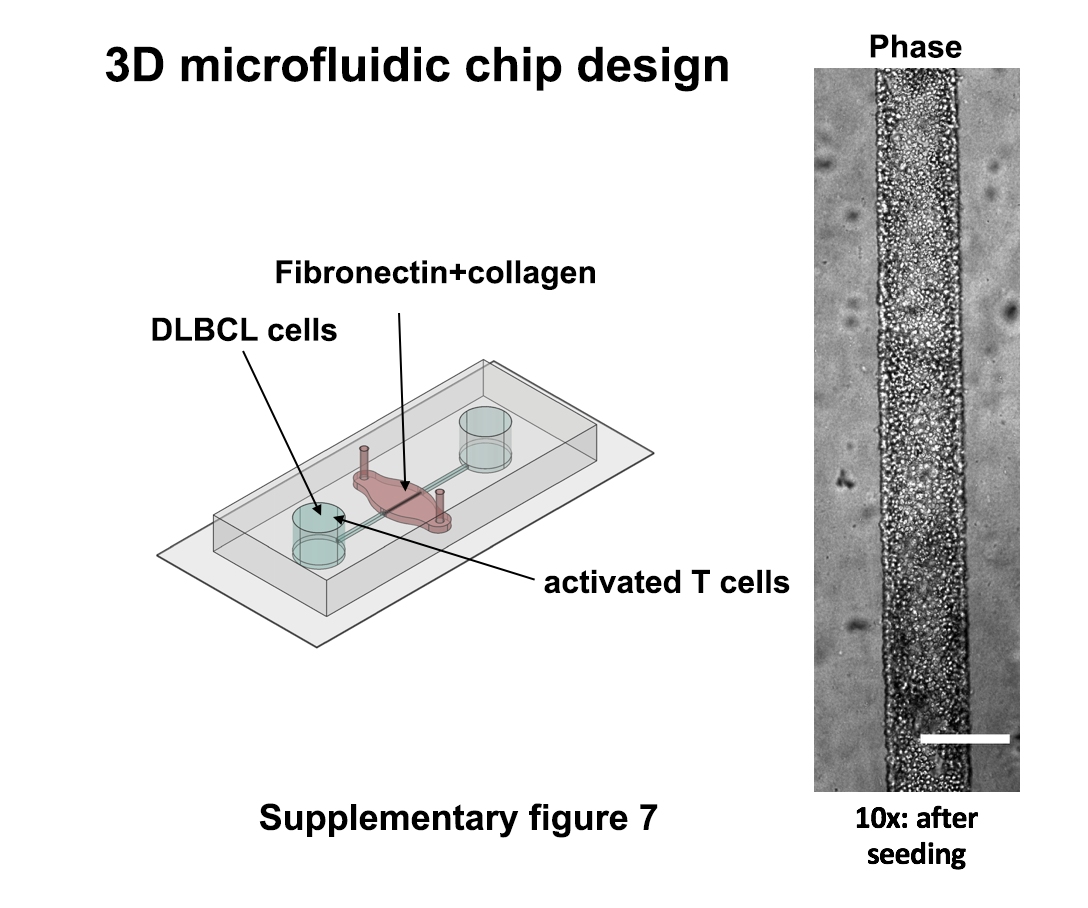

Supplement: Supplementary file 9 — S figure 7 [file 41375_2018_178_MOESM9_ESM.tif]

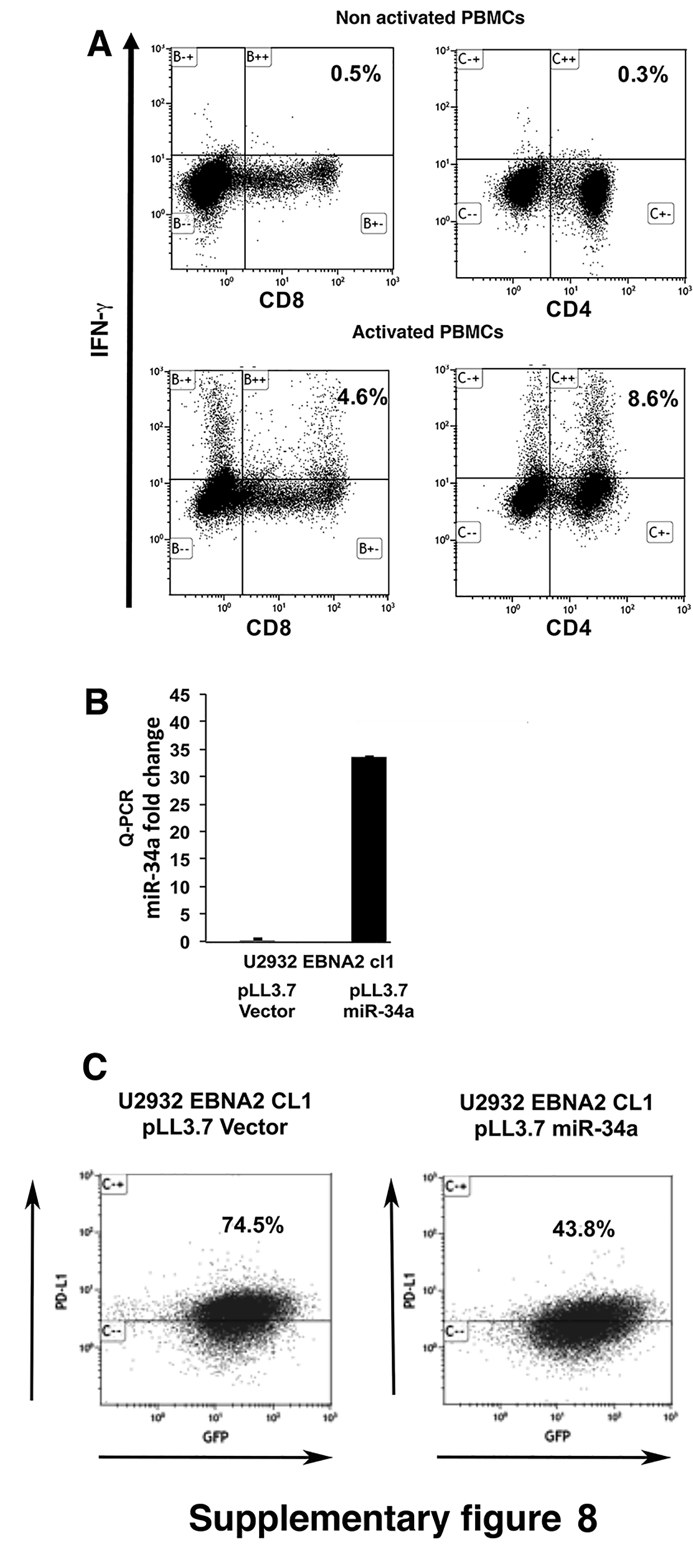

Supplement: Supplementary file 10 — S figure 8 [file 41375_2018_178_MOESM10_ESM.tif]
