# Supplementary material for: Epstein−Barr virus-encoded EBNA2 alters immune checkpoint PD-L1 expression by downregulating miR-34a in B-cell lymphomas
Source: Leukemia. 2018 Jun 26;33(1):132–47. doi: 10.1038/s41375-018-0178-x (PMC6327052; doi:10.1038/s41375-018-0178-x)
Supplement: Supplementary file 11 — S Table 1 [file 41375_2018_178_MOESM11_ESM.docx]

**Supplementary Table 1**:

| **DLBCL^@^** | **EBV** | **EBNA2** | **Type** | **PD-L1**  **staining** | **% PD-L1 positive cells*** | **% intensely stained cells for PD-L1**  **(2+, 3+)*** |
| --- | --- | --- | --- | --- | --- | --- |
| **5** | **+** | **EBNA2+**  **Latency III** | **Non-GC** | **5/5**  **(100%)** | **100§**  **99**  **99** | **86§**  **60**  **64** |
| **6** | **+** | **EBNA2-**  **LatencyII** | **Non-GC** | **6/6**  **(100%)** | **87**  **88**  **78** | **18**  **24**  **20** |
| **10** | **-** | **EBV-** | **Non-GC** | **8/10**  **(80%)** | **35**  **30**  **19** | **1**  **3**  **0** |
| **6** | **-** | **EBV-** | **GC** | **3/6**  **PDL-1+**  **(50%)** | **-** | **-** |

@ A total of 27 DLBCLs (6 GC and 21 non-GC) were included in the study. The classification was done according to Hans Algorithm.

* In three clinical samples representing each DLBCL category, namely EBV neg, EBV+/EBNA2- and EBV+/EBNA2+ ABC DLBCL, the PD-L1 stained sections were digitalized at a 40X magnification using Aperio Scan Scope.

§The percentage positivity was calculated by counting positive cells in three squared areas measuring 50000 μm^2^ in three different samples of each category. On average about 500 cells per region were counted. In the same areas the number of positive cells was determined using the Aperio software IHC Membrane v1. The IHC Membrane Image Analysis algorithm detects the membrane staining for the individual tumor cells in the selected regions and quantifies the intensity and completeness of the membrane staining. +1 intensity is partial membrane staining, +2 is moderate and complete staining and +3 is intense and complete membrane staining.
